# The Effectiveness of a Hybrid Exercise Program on the Physical Fitness of Frail Elderly

**DOI:** 10.3390/ijerph191711063

**Published:** 2022-09-04

**Authors:** Ziyi Wang, Deyu Meng, Shichun He, Hongzhi Guo, Zhibo Tian, Meiqi Wei, Guang Yang, Ziheng Wang

**Affiliations:** 1 Chinese Center of Exercise Epidemiology, Northeast Normal University, Changchun 130024, China; 2 Graduate School of Human Sciences, Waseda University, Tokorozawa 169-8050, Japan; 3 AI Group, Intelligent Lancet LLC, Sacramento, CA 95816, USA; 4 College of Physical Education and Health, Guangxi Normal University, Guilin 541006, China; 5 Advanced Research Center for Human Sciences, Waseda University, Tokorozawa 169-8050, Japan

**Keywords:** frail, Baduanjin, strength training, endurance training, Explainable Artificial Intelligence

## Abstract

Background: Frailty is a serious physical disorder affecting the elderly all over the world. However, the frail elderly have low physical fitness, which limits the effectiveness of current exercise programs. Inspired by this, we attempted to integrate Baduanjin and strength and endurance exercises into an exercise program to improve the physical fitness and alleviate frailty among the elderly. Additionally, to achieve the goals of personalized medicine, machine learning simulations were performed to predict post-intervention frailty. Methods: A total of 171 frail elderly individuals completed the experiment, including a Baduanjin group (BDJ), a strength and endurance training group (SE), and a combination of Baduanjin and strength and endurance training group (BDJSE), which lasted for 24 weeks. Physical fitness was evaluated by 10-meter maximum walk speed (10 m MWS), grip strength, the timed up-and-go test (TUGT), and the 6 min walk test (6 min WT). A one-way analysis of variance (ANOVA), chi-square test, and two-way repeated-measures ANOVA were carried out to analyze the experimental data. In addition, nine machine learning models were utilized to predict the frailty status after the intervention. Results: In 10 m MWS and TUGT, there was a significant interactive influence between group and time. When comparing the BDJ group and the SE group, participants in the BDJSE group demonstrated the maximum gains in 10 m MWS and TUGT after 24 weeks of intervention. The stacking model surpassed other algorithms in performance. The accuracy and precision rates were 75.5% and 77.1%, respectively. Conclusion: The hybrid exercise program that combined Baduanjin with strength and endurance training proved more effective at improving fitness and reversing frailty in elderly individuals. Based on the stacking model, it is possible to predict whether an elderly person will exhibit reversed frailty following an exercise program.

## 1. Introduction

Frailty is a physical illness that increases with age [1,2] and may be accompanied by psychiatric problems such as cognitive impairment [3]. Meanwhile, elderly people who are frail carry a higher risk of falls, hospitalizations, and care home admissions. Frailty has five clinical features, including unexplained body mass loss, fatigue, idleness, slow movement, and weakness, and frailty is diagnosed when three or more of these characteristics are simultaneously present [4,5]. Elderly people who are frail not only carry a higher risk of falls, hospitalizations, and care home admissions [6], but also higher rates of morbidity, mortality, and failure to rescue after major procedures across surgical specialties [7,8,9,10,11,12,13,14,15,16,17,18]. Furthermore, frailty also shows negative effects on health systems and the social economy; nearly half of Medicare spending is attributed to frailty [19]. In the USA, the Netherlands, and Australia, the frail elderly have an average annual healthcare cost that ranges between USD 7500 and 17,500 [20,21,22]. Thus, given the high financial and emotional burden on the families and healthcare systems, affordable treatments for the core diagnostic symptoms of frailty represent a severe unmet medical need.

Fortunately, frailty is reversible and can be prevented, delayed, or even restored to health through specific interventions and personalized health strategies [23,24,25]. An exercise program is an efficient means to enhance the strength of skeletal muscles, improve neuromuscular control, and boost the body’s immunity, which in turn helps the body to recover from frailty [26,27,28]. In terms of type, both strength training and endurance training were shown to be efficient in enhancing neuromuscular control and have a positive impact on cardiorespiratory fitness [29]; as a low-intensity aerobic exercise and Chinese traditional exercise, Baduanjin can improve physical health by working from the inside out [30], and a systematic review has shown its benefits for quality of life, sleep quality, balance, handgrip strength, trunk flexibility, systolic and diastolic blood pressure, and resting heart rate [31]. Moreover, Baduanjin involves soft, slow, simple, and safe movements, which are remarkably suitable for the frail elderly. In addition, since previous research demonstrated that proper training techniques may promote exercise habits in elderly individuals, practices integrating Baduanjin might be considered more successful in inspiring elderly individuals to continue exercising [32].

Although good results were achieved with the frail elderly in the works mentioned above, approximately 42.4–56.3% of them did not experience restored health after the exercise program [33,34]. Fortunately, multi-component exercise has been proven in several studies to have a better effect on frail elderly [25,35,36,37,38,39]. These studies, which incorporate strength training, endurance training, and balance training, were used to improve frailty in older adults. In addition, we proposed a mixed training program of Tai Chi, strength, and endurance that was effective in improving strength, walking speed, and endurance in frail older adults in one of our previous studies [40]. This result raises great confidence that hybrid exercise can better improve the health of the frail elderly. Inspired by previous studies [25,35,36,37,38,39,40], we hypothesized that a hybrid exercise training incorporating Baduanjin could benefit frail older adults by compensating precisely for strength and endurance requirements. Therefore, with the main target of correcting frailty and restoring the physical state in elderly individuals, we added Baduanjin to strength and endurance exercises.

There is a great expectation that personalized medicine will aid in delivering medical care that is more suitable to the individual. However, institutions face difficulties in designing the most appropriate exercise programs due to the complex interaction mechanisms of the human body; in this context, the Explainable Artificial Intelligence (XAI) that we chose to use simulates the clinical treatment process and elucidates its underlying mechanisms of action. Its interpretability could ensure the understanding and trustworthiness of the system [41,42]. Meanwhile, artificial intelligence plays an important role in frailty diagnosis and care [43,44]. Machine learning has also been shown to be effective in screening for frailty and predicting readmission risk in frail individuals [45,46]. Thus, we constructed nine classical models incorporating the characteristics of the frail elderly’s physical fitness status and type of intervention to forecast their frailty after the intervention and thus build exercise regimens for them.

To summarize, the goal of this study was to improve physical fitness and reverse frailty in elderly individuals through constructing a hybrid exercise plan that includes Baduanjin, strength, and endurance training. In addition, we utilized nine machine learning models to forecast frailty according to basal physical fitness and distinct interventions. We established the following study hypotheses based on the findings of past investigations: (1) initial physical fitness and intervention programs can be predictive of frailty overcome results; (2) a hybrid exercise program that integrates Bajuanjin, strength, and endurance training can enhance physical fitness and overcome frailty in the elderly.

## 2. Materials and Methods

### 2.1. Participants

In order to ensure the safety of procedures and to avoid bias in results, participants were included in this study who met the criteria as follows: Inclusion Criteria: (1) age over 65 years old; (2) meet criteria for frailty, as defined by Fried et al. [5]; (3) no other training within 6 months. Exclusion Criteria: (1) a history of neurological or muscular disorders; (2) having joint discomfort and significant muscle and bone injuries that prevent them from walking normally; (3) having a heart or respiratory illness; and (4) practising any other training courses frequently during the time of the trial. The determination of the sample size was supported by previous intervention research on elderly people who were frail [40]. This study had an effect size of 0.53, and it had 80% power with an alpha level of 0.01. Additionally, it had a dropout frequency of 20%.

### 2.2. Study Design

#### 2.2.1. Experimental Arrangement

**Figure 1:**
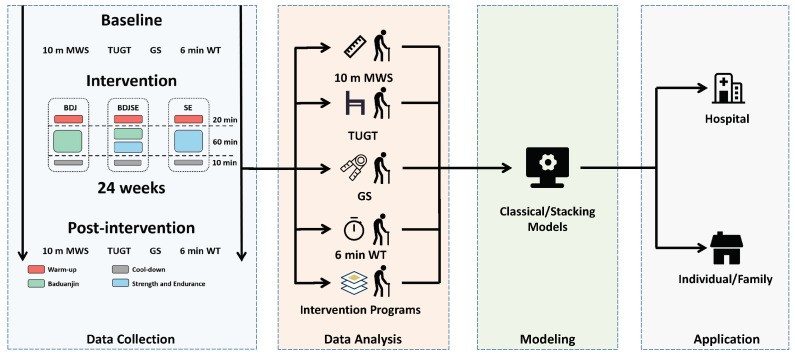
System design; all of the subjects were randomly assigned to one of three intervention groups: BDJ, BDJSE, or SE. At the beginning of the study and at 24 weeks, the subjects’ 10-meter maximum walk speed (10 m MWS), timed up-and-go test (TUGT), grip strength, and 6 min walk test (6 min WT) were evaluated.

This experiment was performed in a randomized, double-blinded fashion, focusing on the frail elderly to enhance their physical fitness and reverse the frailty. The experiment and application system are shown in Figure 1. Experiments were carried out every Monday, Wednesday, and Friday for a total of 24 weeks. Two Baduanjin instructors were employed: one was a nationwide societal professional coach with more than 8 years of experience as a training instructor; the other instructor was a specialist Baduanjin coach who had obtained a national Baduanjin instructor industry accreditation. All subjects for the study were recruited from the community in Changchun, China. We used the random number table method to randomly assign participants. Subjects were randomized into three groups: the Baduanjin intervention group (BDJ), the Baduanjin, strength, and endurance group (BDJSE), or the strength and endurance group (SE). In March of 2019, after the review and approval of the study protocol by the Ethical Committee of Northeast Normal University, each participant provided their signature on a permission form, indicating that they had read and understood the study’s procedures (approval number: NC2018091504). Between the 1st of March and the 30th of September, 2019, measurements and data collection were carried out. It was requested that the subjects avoided engaging in any type of physical exercise other than their typical routine of training.

#### 2.2.2. Intervention

The subjects participated in three separate intervention programs, which comprised endurance training, strength training, and Baduanjin. At the beginning of the intervention, each group spent 20 min warming up with various exercises. The Baduanjin training in the BDJ group lasted for 60 min, whereas the Baduanjin training in the BDJSE group lasted for 30 min and then continued for 30 min of strength and endurance exercise. The SE group completed a strength and endurance workout that lasted for 60 min. Ahead of the end point of the intervention, there was a 10-min cool-down period for all groups. The following is an outline of the training procedure.

(1) Baduanjin: The exercise regimen of Baduanjin was divided into two phases: the first phase continued for 8 weeks, while the second phase continued for 16 weeks. During the first phase of the project, one group was assigned to perform the intervention three times, while another group, the BDJSE group, was assigned to perform the exercise only once. In the next stage, the number of replicates was three and two for the BDJ and BDJSE groups, respectively.
(2) Strength Training: This consisted of three training phases and included five exercises to improve cardiorespiratory fitness and muscle strength [47]. The five movements included three upper-body movements and two lower-body movements. The three upper-body movements were seated rowing, reverse grip curls, and bicep curls, and the two lower-body movements were calf lifts when seated and hip adduction exercises. Elastic bands were utilized in each and every one of the strength workouts. The intensity of the exercise could be determined by the elastic band color. The training consisted of three distinct phases that were repeated every 8 weeks. Phase I aimed to better acclimate subjects to the high-intensity exercise in Phases II and III by using light loads (40–60% of 1RM) and high repeats (12–20), while simultaneously increasing muscle power and muscle endurance by accomplishing 2–4 rounds of training workouts. The second phase of the program was designed to induce muscle growth and improve the muscle mass to fat mass ratio by continuously raising the load to ultimate capacity (60.0–80.0% of 1RM) with 5–12 repeats and 2–4 rounds. The training protocols were intended to achieve these goals. The third phase was intended to optimize the development of strength and also encourage the growth of muscular tissue by utilizing a greater load (70–85% of 1RM) for 5–8 repeats over 2–4 rounds. The SE group would finish four rounds, while the BDJSE group would finish two rounds, with a break of between 2 and 3 min after each round.
(3) Endurance Training: We monitored the subjects’ heart rates during the exercise period using a heart rate monitor (MYZONE MZ-3, China). The exercise was conducted via continuous walking on an artificial track. In this investigation, the target heart rate was adapted separately for each subject based on the baseline measure. Exercise level was progressively elevated from 50% of baseline heart rate capacity (first 12 weeks) to 80% (the following 12 weeks) [48]. The SE group undertook 30 min of endurance walking exercise, while the BDJSE group accomplished 15 min. In all exercises, at least two medical staff accompanied the training, and the training was promptly terminated if the subjects became uncomfortable.

### 2.3. Assessment of Frailty

Fried frailty assessment criteria is widely used in the Asia-Pacific region. One previous study showed that this criterion has better validity and feasibility among older adults in the Chinese community [49]. Thus, the Fried frailty criteria [5] were employed in this work to identify all levels of perceived frailty. According to these criteria, aged people are considered to have frailty when at least three of the following five phenotypic characteristics exist.

(1) Unconscious weight loss: Participants were asked whether their weight had decreased by more than 4.5 kg (or 5% of body weight) without intention in the past year.
(2) Self-reported fatigue: Participants were asked how often they were too exhausted to participate in any activity that required their full engagement for more than 2 days in a week.
(3) Grip strength: Subjects’ grip strengths were determined by utilizing a calibrated Jamar Hydraulic Hand Dynamometer (model SH5001, Saehan Corp, Masan, Korea, 2017). Every person was given three chances to be evaluated, and their highest score was counted. The grasp was examined to determine if males weighed less than 26 kg and females weighed less than 18 kg.
(4) The walking speed: The 10 meter walk speed of the subjects was recorded. Older people were judged frail if their walking speed was lower than or equal to 1 m/s.
(5) Low level of physical activity: The level of physical activity of individuals was determined by the Physical Activity Scale for the Elderly in the Chinese population (PASE-C) [50]. Low physical activity was defined in men as a cut-off value of less than 383 calories per week and in women of less than 270 calories per week, respectively.

### 2.4. Assessment of Physical Fitness

The physical performance of the participants was evaluated pre- and post-intervention by utilizing the 10-meter maximum walk speed (10 m MWS), the timed up-and-go test (TUGT), grip strength, and the 6 min walk test (6 min WT). Following the collection of primary information, we computed the split-half reliability of the participants’ initial testing values to assess reliability. The findings indicated that all four test techniques were reliable (r = 0.82, *p* < 0.001; half-score reliability). It was discovered that the 10 m MWS had strong validity in older persons since there was a significant correlation between the item and alterations in frailty [51]. The TUGT is an easy test that requires no specialized device and was proven to have a high degree of validity for assessing agility (r = 0.63) [52]. An increased risk of impairment, mortality, and illness is associated with lowering grip strength, which is a feature of frailty in old age [53,54]. The research conducted by Syddall et al. indicated that grip strength was significantly related to measures of frailty and precisely mirrored overall muscle strength (r = 0.69) [55]. In older people, frailty can be indicated by multiple variables, including low levels of fitness and stamina, as well as decreased neuromuscular function. The 6 min WT was found to have excellent validity (r = 0.77) in assessing indirectly the endurance quality (maximal oxygen consumption) of elderly individuals [56]. The following describes the test’s features:

(1) 10 m MWS: Subjects performed two 50-m walking exercises as quickly as possible in a calm testing setting, and the time to cover 2.5 to 12.5 m was calculated to ensure the steady status of data. The highest value was utilized in the study.
(2) TUGT: Subjects were seated in a conventional chair 45 cm in height and, when prompted by the research assistant, stood up and performed a 3 meter circumference walk around the room as quickly as possible before returning to their seat.
(3) Grip strength: Grip strength was measured utilizing a calibrated Jamar Hydraulic Hand Dynamometer (model SH5001, Saehan Corp, Masan, Korea, 2017). In a standing position, subjects conducted three grip strength assessments, and the best score was considered the test result.
(4) 6 min WT: The 6 min WT was utilized to evaluate the endurance of the subjects. The test was conducted on a 30-m, enclosed, level promenade. Along the promenade, signs were set every 3 meters, and turn signals were established at each end. Individuals were urged to cover the greatest distance possible along the promenade.

Prerequisites for any examination of a participant’s motor abilities included, firstly, that the test participants be attired in appropriate athletic clothing and footwear. They each carried out the test on their own. Second, each subject was required to become familiar with the procedure involved in the experiment in preparation; third, the subjects were required to perform a warm-up to prevent injuries; ultimately, if the subject experienced bodily distress, stress connected to body posture, or ecologic discomfort, they were asked to notify the researchers. The test could be canceled at any moment.

### 2.5. Data Analyses

When attempting to determine the post-experimental frailty state of elderly individuals who were already frail, researchers used a total of eight classical machine learning classification models. The 10 m MWS, grip strength, TUGT, 6 min WT, and three intervention types before the intervention were utilized as characteristics in the creation of the data. Labels for the dataset included whether or not the subjects were feeble after the intervention. The effectiveness of the testing measures was judged according to their levels of accuracy, recall, and prediction, as well as their areas under the curve (AUC). In order to obtain an accurate assessment of the performance of the model, we carried out the stratified 10-fold cross-validation 100 times. In the beginning, traditional machine learning modeling was carried out with the assistance of the LightGBM Classifier (LGBM) [57], Gradient Boosting Classifier (GBC) [58], XGBoost Classifier (XGB) [59], Extra Tree Classifier (ETC) [60], Decision Tree Classifier (DT) [61], Random Forest Classifier (RF) [62], Linear Discriminant Analysis (LDA) [63], and Logistic Regression (LR) [64]. Following this, the three models that had the best overall effectiveness within these records were chosen for stacking modeling. For this particular investigation, the process of stacking was carried out by integrating multiple classifiers that were produced by various learning algorithms $L_{1},\ldots ,L_{n}$ on a single dataset *S*. This dataset comprised examples that had the form $S_{i}$ = ($x_{i}$, $y_{i}$), where $x_{i}$ represents the characteristic vectors and $y_{i}$ represents the classifications. In the initial step of the process, a group of base-level classifiers known as $C_{1}$, $C_{2}$, and $C_{3}$ were developed, with $C_{i}$ = $L_{n}$ (*S*). The second stage consisted of learning a meta-level classifier that comprised the outcomes of the base-level classifiers. To produce a training set for the purpose of learning the meta-level classifier, a cross-validation process was carried out, in which each of the base-level learning algorithms was applied to the whole dataset. We retained only one sample for examination, such as $\forall i=1,\ldots ,n:\forall k=1,\ldots ,N:C_{k}^{i}=L_{k}\left(S-s_{i}\right)$, and then used the learned classifiers to create projections for $S_{i}$, as in Equation (1):

(1) $${\hat{y}}_{i}^{k}=C_{k}^{i}\left(x_{i}\right),$$

where the meta-level dataset comprised examples of the form ((${\hat{y}}_{i}^{1}$, …, ${\hat{y}}_{i}^{n}$), $y_{i}$), the characteristics were the expectations of the base-level classifiers, and the class was the appropriate category for the example considered. By calculating SHAP values (SHapley Additive exPlanations) [65], we were able to determine which characteristic provided the maximum anticipation of change in frailty. SHAP is a game-theoretic method to interpret the outcome of any machine learning model. SHAP values could measure the impact that each feature provides to the estimate provided by the model, as in Equation (2):

(2) $$\phi_{j}=\sum_{S_{F}\subseteq F\backslash \left\{j\right\}}\frac{|S_{F}\left|!(|F|-\right|S_{F}\left|-1\right)!}{|F|!}\left[f_{S_{F}\cup \left\{j\right\}}\left(x_{S_{F}\cup \left\{j\right\}}\right)-f_{S_{F}}\left(x_{S_{F}}\right)\right],$$

where *x* indicates input features’ value, *j* denotes a certain feature (out of total features *F*), $S_{F}$ is entire subsets without *j*, and $|S_{F}|$ indicates the dimension of $S_{F}$. In this study, the SHAP “TreeExplainer” algorithm was used to evaluate the feature contribution of predicting reversing frailty, the model $f_{S_{F}\cup \left\{j\right\}}$ was trained with feature *j* present, and another model $F_{S_{F}}$ was trained with feature *j* withheld. Data analysis and visualization used Python 3.8.1 in this study.

### 2.6. Statistical Analyses

We used SPSS 25.0 to analyze the demographic variables of participants at the baseline and the effectiveness of the three intervention programs on participant improvement. The Shapiro–Wilk test was employed to examine whether the distribution was normal, and logarithmic transformation was performed for data that lacked a normal distribution. The mean ± standard deviation (SD) were computed, and values were applied to express continuous variables, whereas other values were used to express categorical variables. At the beginning of the study, the demographic factors of the subjects were examined utilizing chi-square tests and one-way ANOVA. The two-way repeated-measures ANOVA was used to determine how much of an impact each of the three intervention regimens had on the individuals’ improvements in their 10 m MWS, TUGT, grip strength, and 6 min WT. When multiple comparisons were made, Bonferroni post-hoc testing was employed. When *p* < 0.05, the result was deemed statistically significant.

## 3. Results

### 3.1. Participants

In this study, 103 females and 100 males out of a total of 271 individuals passed the selection process to become one of the 203 subjects who were chosen. The research was finished with the participation of 171 people (92 females and 79 males). There were a total of 15 people who dropped out of the training by their own choice, while 17 people dropped out of the training because of illness. The demographic profile of the subjects at the start of the study is presented in Table 1. The results of the Shapiro–Wilk test showed that age (*p* = 0.334), stature (*p* = 0.452), and body mass (*p* = 0.165) were normally distributed in the three groups. In terms of demographics, the groups showed no significant differences from one another.

### 3.2. Two-Way Repeated-Measures ANOVA Results for Physical Fitness

**Figure 2:**
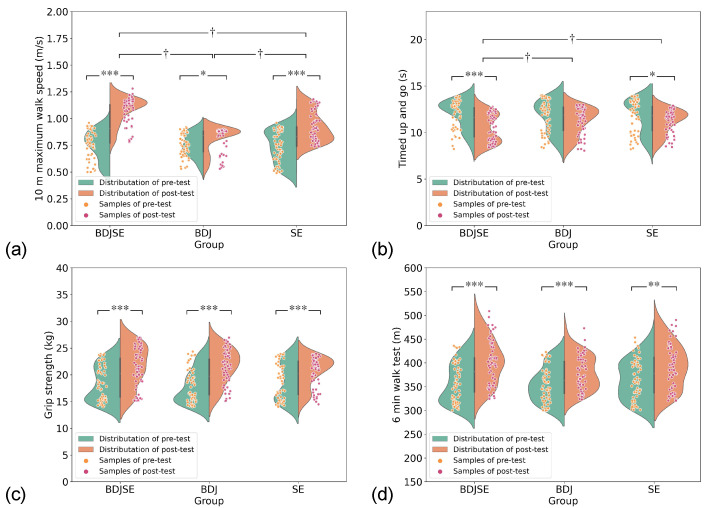
The scatter plot with a rotated kernel density plot on every side. It illustrates the physical capability of the frail elderly at the start and after the intervention. (**a**) 10 m MWS, (**b**) timed up-and-go test, (**c**) grip strength, (**d**) 6 min WT. BDJ denotes Baduanjin group; BDJSE denotes Baduanjin, strength, and endurance group; SE denotes strength and endurance group. * *p* < 0.05, ** *p* < 0.01, *** *p* < 0.001. For the significance of intragroup variations, † shows a significant difference among groups († < 0.05).

Figure 2 and Table 2 illustrate the starting and post-intervention findings. The results of the Shapiro–Wilk test showed that 10 m MWS (*p* = 0.517), grip strength (*p* = 0.184), and TUGT (*p* = 0.257) were normally distributed in the three groups. However, 6 min WT demonstrated a non-normal distribution in the three groups. When logarithmic transformation was applied to it, the data showed a normal distribution. Two-way repeated-measures ANOVA data confirmed that there was a significant interaction impact of group × time in 10 m MWS (*p* < 0.001, partial $\eta^{2}$ = 0.259) and TUGT (*p* = 0.011, partial $\eta^{2}$ = 0.117). Simple effects analysis revealed that 10 m MWS and TUGT were significantly enhanced following the intervention. Post-hoc testing demonstrated that individuals in the BDJSE group had significantly better 10 m MWS than the BDJ group (*p* < 0.001) and SE group (*p* < 0.001) and better TUGT than the BDJ group (*p* = 0.019) and SE group (*p* = 0.038) in 24 weeks. Nevertheless, we observed no significant interaction influence of grouping time on grip strength and 6 min WT. The major effect demonstrated that, before and after the intervention, the grip strength and 6 min WT experienced a significant increase.

### 3.3. Results of Machine Learning Model Classification

In addition, we reassessed the participants’ frailty status at 24 weeks. The results showed that 25 participants (30.1%) in the BDJ group progressed from a frail condition to a non-frail condition, 33 participants (39.8%) in the BDJSE group progressed from a frail condition to a non-frail condition, and 25 participants (30.1%) in the SE group progressed from a weak condition to a non-frail condition, for a total of 83 participants (48.5%).

We used intervention types and participants’ 10 m MWS, TUGT, grip strength, and 6 min WT at baseline as features and frailty and non-frailty at 24 weeks as the label. We used eight classical machine learning models and selected three of them with the best performance for stacking. The three classification models, Linear Discriminant Analysis, Logistic Regression, and Random Forest Classifier were stacked to create the first layer mode to construct the super features. In the second layer, we used Logistic Regression, inputting the super features and labels into the second layer model for training. The stacking model obtained the best accuracy as 75.5 ± 10.0% and best precision as 77.1 ± 12.3%, as shown in Table 3. However, Linear Discriminant Analysis had the best F1-score (71.3 ± 10.8%) and best recall (73.7 ± 15.1%).

When we compared the effectiveness of the various models, we found that the stacking model provided the greatest results in terms of accuracy and precision. The first layer of the stacking model consisted of Linear Discriminant Analysis, Logistic Regression, and Random Forest Classifier. The second layer of the stacking model consisted of Logistic Regression.

**Figure 3:**
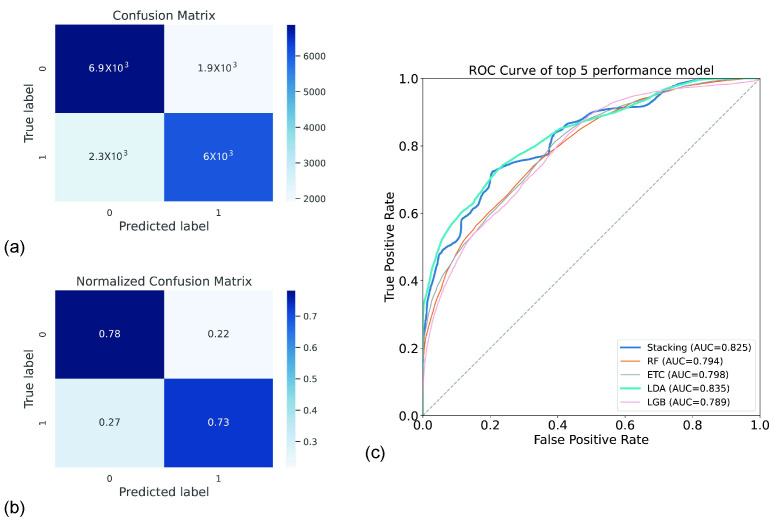
The confusion matrix and the ROC curve of stacking. (**a**) The confusion matrix; (**b**) the normalized confusion matrix. The performance of the model to determine whether a participant is an elderly individual at risk of frailty is displayed in the figure. (**c**) The Receiver Operating Characteristic (ROC) curve of the five best-performing models. Random Forest Classifier (RF), Extra Tree Classifier (ETC), Linear Discriminant Analysis (LDA), and LightGBM Classifier (LGB) are the abbreviations for other classification methods.

In addition, we evaluate the model performance using a confusion matrix and Receiver Operating Characteristic (ROC) curve. The confusion matrix showed the model’s anticipation of the frailty state, and the normalized confusion matrix could visualize the prediction accuracy of the model. The elements of the sub-diagonal line in Figure 3a for the confusion matrix indicated the number of predicted categories that were the same as the true category and the elements of the sub-diagonal in Figure 3b. The mean precision of the model’s estimation for every label, as indicated by the normalized confusion matrix, was 78%, with 73% being the value for the other label. The ROC curve of the model for estimating the performance of the model is depicted in Figure 3c. The horizontal coordinate demonstrates the false positive frequency, while the vertical coordinate demonstrates the frequency of true positives. The model with the best (AUC) was the Linear Discriminant Analysis model, which had a value of 0.835.

### 3.4. Contribution of Each Feature

**Figure 4:**
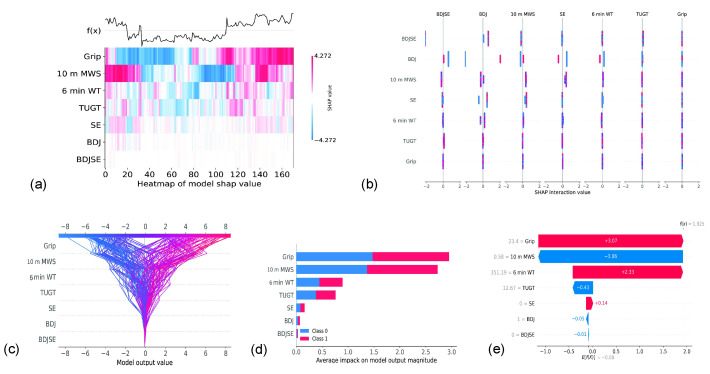
A plot that provides an overview of the SHAP values for every feature. The various characteristics are denoted along the *y*-axis. (**a**) illustrates the heat map of the various characteristic SHAP values, in which the *x*-axis depicts the series of the sample, red denotes a positive effect, blue denotes a negative effect, and the darker the color, the greater the extent to which it has an effect. The output is denoted by the function *f*(*x*) (before activation function). It is shown in the figure that the greatest essential characteristic shows a positive impact if the output also appears positive, and it demonstrates a clear boundary (threshold) in the characteristics; (**b**) shows the interaction SHAP values for various characteristics; (**c**) provides an outline of the characteristics that are highly essential to the model and the manner in which the model learned the outcome of every characteristic for each sample, with the *x*-axis denoting the model predicted values; (**d**) demonstrates the mean absolute SHAP value of each characteristic, with the *x*-axis denoting the average SHAP value; (**e**) depicts characteristics that have a role in displaying the model result from the fundamental value of a specific sample, with the SHAP value denoted along the *x*-axis. According to the previous situation, the grip strength demonstrated the most positive effect for predicting the frailty state and the 10 m MWS demonstrated a negative effect for predicting the frailty state.

Figure 4 demonstrates the contribution of each feature by SHAP values. It is possible to utilize the SHAP value of a characteristic to describe the model since it indicates the value that each feature contributes to the model. Figure 4d clearly illustrates this, as grip strength showed the greatest contribution to physical fitness and SE showed the greatest contribution in the three intervention types. The features could interact with each other, as shown in Figure 4b. Clearly, we were able to find the best combination of features to optimize the model performance by utilizing different combinations of features.

## 4. Discussion

This study is unique in considering the use of combined physical activities that address the characteristics of the frail elderly by integrating Baduanjin exercises with strength and endurance training programs and predicting frailty pre- and post-intervention in a clinical trial by using AI. The results showed that all of the experimental groups exhibited some degree of physical progress in terms of their strength, velocity, and endurance after 24 weeks of training. Among them, the mixed-exercise program group showed the best physical improvement in subjects in the 10 m MWS and TUGT.

In addition, the combined workout regimen had the best effectiveness in counteracting frailty, with 46.5% of frail elderly individuals recovering from a non-frail status as a result of participating in the program.

In addition, we incorporated XAI into this study. The stacking model obtained an average accuracy of 75.5% by simulating clinical application scenarios, with physical ability at baseline and intervention type as model attributes used as inputs, and frailty reversed was used as a result. Additionally, we found that 10 m MWS and grip strength have high contributions and identifiability: elderly individuals who are frail and have higher 10 m MWS and grip strength have a greater chance of recovering from frailty. As a result, increasing the grip strength and 10m distance of elderly individuals who are frail may be able to maximize the efficacy of the intervention.

There was a significant interaction effect in 10 m MWS, as 1.08 m/s (BDJSE) > 0.93 m/s (SE) > 0.82 m/s (BDJ), and TUGT, as 11.21 s (BDJ) > 11.19 s (SE) > 10.47 s (BDJ), at 24 weeks. This is consistent with several previous studies in which multicomponent exercise improved gait capacity and agility in frail older adults [35,38]. The reasons that a combined training schedule had the best effectiveness in 10 m MWS and TUGT are as follows. A multi-component exercise program is a good approach because it allows aged people who are frail to benefit from a variety of training modalities to address any weaknesses they may have. This is a useful method since aged individuals are more likely to experience frailty while they age [28]. Second, the Baduanjin exercise can help participants to improve the sensorimotor ability of their legs [66], and it also has additional benefits for the strength of the muscles in the legs [31]. Both of these benefits can be gained from performing the exercise. As a consequence, the endurance training that is performed in the style of walking compensates for the comparatively slow movement attributes of Baduanjin and assists in improving the participants’ capacity to walk under natural settings. Ultimately, DBJSE hybrid exercise enhanced the walking speed and Baduanjin could effectively improve the lower balance function [66]. Therefore, participants in the BDJSE group had significantly better 10 m and grip strength than those in the other two groups.

In this study, participants’ grip strength was significantly improved at 24 weeks, but the interaction effect was not significant. This is consistent with a previous study in which the intervention protocol consisted of strength training, endurance training, and balance training [35]. Participants in the SE group had the greatest average grip strength (21.63 kg (SE) > 21.58 kg (BDJSE) > 20.60 kg (BDJ)) and maximum improvement ((3.70 kg (SE) > 3.14 kg (BDJSE) > 1.91 kg (BDJ)). Although Baduanjin can improve grip strength, it seems to be more effective for young people [66]. The BDJSE group included the training content of the SE group, but the overall load was only half that of the SE group, so participants in the BDJSE group showed less improvement.

The 6 min WT also did not show a significant interaction, but, fortunately, all three groups of subjects showed a significant increase. This is consistent with a previous study showing that multicomponent exercise improves aerobic capacity in frail older adults [67]. A possible reason is that Baduanjin can increase lung capacity and lower the resting heart rate [31], and, by combining endurance training with walking as a form of exercise, the endurance improvement effect was increased.

Our study had the desired effect of reversing frailty and improving the exercise capacity of frail older adults through a 24-week mixed exercise program. In the future recovery of frail older adults, a mixed exercise program may be more effective in improving health, such as the Baduanjin exercise hybrid strength and endurance training proposed in this study.

Although our study yielded promising results, limitations still remain. First, we did not classify the severity of frailty, and, in future studies, we will further refine the intervention content. Second, we did not have strict control over the intensity, only controlling the duration of the training. Finally, we strongly believe that there is a potential correlation between physical fitness and frailty reversal in frail aged people. Based on this relationship, in future studies, we will integrate more data to accomplish more precise medical clinical assistance.

## 5. Conclusions

Our findings indicate that a combined exercise regime that combines Baduanjin, strength training, and endurance training could effectively increase physical performance, particularly 10 m MSW and TUGT, as well as reverse frailty in the frail elderly. Additionally, the stacking model had the best performance to predict the reversal of frailty.

## Figures and Tables

**Table 1:** Baseline demographic characteristics of the participants.

Items	BDJ ^1^ (*n* = 57)	BDJSE ^2^ (*n* = 57)	SE ^3^ (*n* = 57)	*p* Value
Sex (male/female)	28/29	27/30	25/32	0.736
Age (years)	71.84 ± 3.77	70.65 ± 3.73	70.74 ± 3.52	0.163
Stature (cm)	165.83 ± 6.77	163.41 ± 7.58	165.54 ± 8.22	0.182
Body mass (kg)	64.53 ± 5.59	62.97 ± 7.11	63.05 ± 6.88	0.378

^1^ Baduanjin group; ^2^ Baduanjin, strength, and endurance group; ^3^ strength and endurance group.

**Table 2:** Two-way repeated-measures ANOVA results for each group at baseline and 24 weeks for the test metrics.

Parameters	BDJ ^1^ (*n* = 57)		BDJSE ^2^ (*n* = 57)		SE ^3^ (*n* = 57)		Group × Time ^#^
	Baseline	24 Weeks	Baseline	24 Weeks	Baseline	24 Weeks	*p*-Value
10 m MWS (m/s)	0.75 ± 0.11	0.82 ± 0.12 ^†,^*	0.75 ± 0.14	1.08 ± 0.12 ^†,^***	0.73 ± 0.13	0.93 ± 0.14 ^†,^***	0.000
TUGT (s)	11.76 ± 1.67	11.21 ± 1.48 ^†^	12.01 ± 1.50	10.47 ± 1.51 ^†,^***	11.90 ± 1.65	11.19 ± 1.29 ^†,^*	0.041
grip strength (kg)	18.69 ± 3.50	20.60 ± 2.77 ***	18.44 ± 3.28	21.58 ± 3.82 ***	17.93 ± 3.14	21.63 ± 3.26 ***	0.080
6 min WT (m)	355.25 ± 37.02	380.06 ± 36.55 ***	357.75 ± 42.01	403.21 ± 47.61 ***	365.07 ± 42.11	392.45 ± 47.49 **	0.154

Means × SD are used to depict all the data; ^#^ analysis of two-way repeated-measures ANOVA; ^†^ significant difference between groups (*p* < 0.05); * *p* < 0.05, ** *p* < 0.01, *** *p* < 0.001 significant difference between baseline and post-intervention. ^1^ Baduanjin group; ^2^ Baduanjin, strength, and endurance group; ^3^ strength and endurance group.

**Table 3:** Model performance evaluation results.

Models	Accuracy	Precision	Recall	F1
Decision Tree (%)	66.3 ± 11.2	65.4 ± 12.8	65.3 ± 16.2	65.1 ± 12.1
GDB Classifier ^1^ (%)	66.7 ± 10.6	66.7 ± 13.2	65.5 ± 16.3	64.5 ± 12.5
XGB Classifier ^2^ (%)	68.8 ± 10.9	70.5 ± 13.2	65.1 ± 15.4	66.7 ± 12.2
LGBM Classifier ^3^ (%)	69.2 ± 10.6	70.4 ± 13.2	68.0 ± 15.6	68.0 ± 11.9
Extra Tree Classifier (%)	69.7 ± 10.2	70.5 ± 12.7	68.1 ± 15.6	68.0 ± 12.0
RF Classifier ^4^ (%)	70.3 ± 10.5	71.5 ± 13.4	66.7 ± 16.3	67.4 ± 12.2
Logistic Regression (%)	73.7 ± 10.3	74.9 ± 12.5	71.3 ± 16.0	72.1 ± 11.1
LDA Classifier ^5^ (%)	75.3 ± 10.3	76.2 ± 12.3	**73.7 ± 15.1**	**74.0 ± 11.6**
**Stacking** (%)	**75.5 ± 10.0**	**77.1 ± 12.2**	72.8 ± 15.0	73.9 ± 11.3

^1^ Gradient Boosting Classifier, ^2^ XGBoosting Classifier, ^3^ LightGBM Classifier, ^4^ Random Forest Classifier, ^5^ Linear Discriminant Analysis.

## Data Availability

The data used and/or analyzed during the current study are available from the corresponding author on reasonable request.
